# Anatomical-Molecular Distribution of EphrinA1 in Infarcted Mouse Heart Using MALDI Mass Spectrometry Imaging

**DOI:** 10.1007/s13361-017-1869-7

**Published:** 2018-01-05

**Authors:** Stephan Lefcoski, Kimberly Kew, Shaun Reece, Maria J. Torres, Justin Parks, Sky Reece, Lisandra E. de Castro Brás, Jitka A. I. Virag

**Affiliations:** 1 Department of Physiology, Brody School of Medicine, East Carolina University, Greenville, NC 27834 USA; 2 Department of Chemistry, East Carolina University, Greenville, NC 27834 USA; 3 East Carolina Diabetes and Obesity Institute, East Carolina University, Greenville, NC 27834 USA

**Keywords:** MALDI/IMS, EphrinA1, Myocardial infarction

## Abstract

EphrinA1 is a tyrosine kinase receptor localized in the cellular membrane of healthy cardiomyocytes, the expression of which is lost upon myocardial infarction (MI). Intra-cardiac injection of the recombinant form of ephrinA1 (ephrinA1-Fc) at the time of ligation in mice has shown beneficial effects by reducing infarct size and myocardial necrosis post-MI. To date, immunohistochemistry and Western blotting comprise the only experimental approaches utilized to localize and quantify relative changes of ephrinA1 in sections and homogenates of whole left ventricle, respectively. Herein, we used matrix-assisted laser desorption ionization mass spectrometry imaging (MALDI-MSI) coupled with a time-of-flight mass spectrometer (MALDI/TOF MS) to identify intact as well as tryptic fragments of ephrinA1 in healthy controls and acutely infarcted murine hearts. The purpose of the present study was 3-fold: (1) to spatially resolve the molecular distribution of endogenous ephrinA1, (2) to determine the anatomical expression profile of endogenous ephrinA1 after acute MI, and (3) to identify molecular targets of ephrinA1-Fc action post-MI. The tryptic fragments detected were identified as the ephrinA1-isoform with 38% and 34% sequence coverage and Mascot scores of 25 for the control and MI hearts, respectively. By using MALDI-MSI, we have been able to simultaneously measure the distribution and spatial localization of ephrinA1, as well as additional cardiac proteins, thus offering valuable information for the elucidation of molecular partners, mediators, and targets of ephrinA1 action in cardiac muscle.

**Electronic supplementary material:**

The online version of this article (10.1007/s13361-017-1869-7) contains supplementary material, which is available to authorized users.

**Graphical Abstract:**
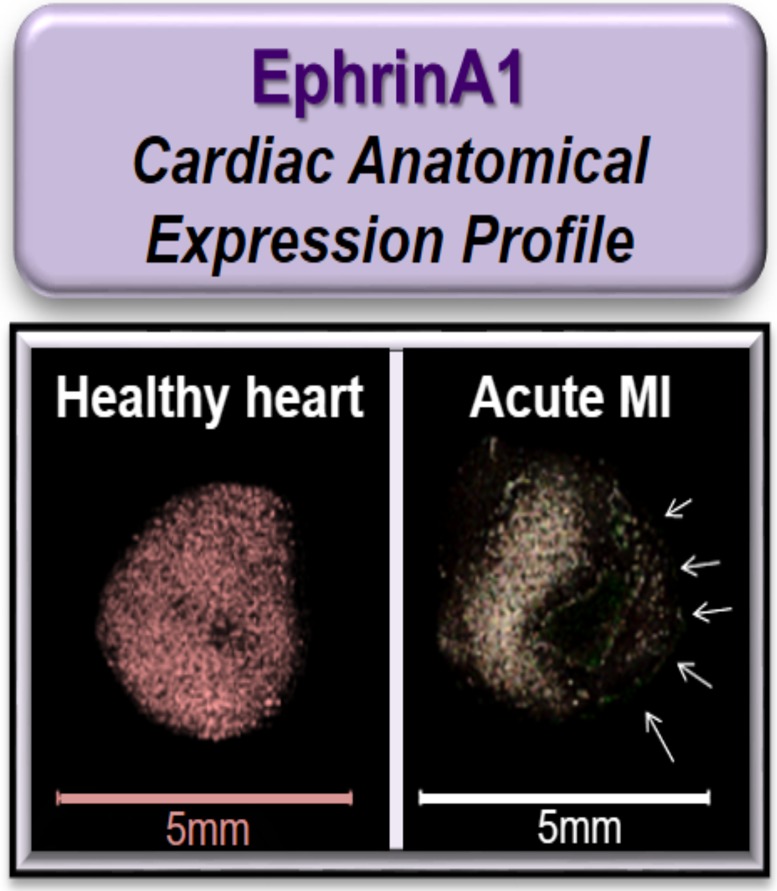
ᅟ

## Introduction

Heart disease represents a staggering burden in the US in terms of survival rates and healthcare costs [1, 2]. High rates of mortality associated with myocardial infarction (MI), and especially with recurrent MIs, indicate the need for novel diagnostic and therapeutic methods. EphrinA1 is a tyrosine kinase receptor expressed in the cellular membrane of cardiomyocytes. After myocardial injury, cardiomyocytes become necrotic or damaged, which leads to a substantial reduction in ephrinA1 protein levels. Immunohistochemical studies have shown that cell surface expression of the endogenous ephrinA1 ligand decreases in injured cardiomyocytes during an MI [1, 2]. However, a recent study showed that intra-cardiac injection of recombinant ephrinA1-Fc post-MI reduced infarct size, cardiomyocyte necrosis, chamber dilation, and left ventricular free wall thinning in mice [2]. The beneficial effects on cardiac function observed with ephrinA1-Fc treatment indicate that this molecule is a promising, novel therapeutic for MI treatment. However, the precise molecular mechanism(s) by which exogenously administered ephrinA1-Fc prevents cardiomyocyte death post-MI remain unknown.

With intra-myocardial levels of ephrinA1 following an MI being positively correlated with favorable outcomes, insights into ephrinA1 intracellular signaling pathways suggest that blocking apoptosis and inflammation, as well as promoting autophagy, could represent potential mechanisms of action [2, 3]. A more in-depth understanding of the molecular distribution of ephrinA1 and its mediators in cardiac tissue is vital for the identification of targets that could minimize cardiomyocyte death and prevent heart failure following MI. Previous studies have provided qualitative information on the molecular distribution of ephrinA1 via immunohistochemistry, as well as relative quantitative data by Western blotting of tissue homogenates [1]. However, the large number of chromogens or fluorochromes that can cause interference, as well as their different subcellular localization, significantly limits the number of proteins that can be identified simultaneously using immunohistochemical techniques; and Western blotting per se fails at deciphering a protein’s true anatomical distribution. Matrix-assisted laser desorption ionization mass spectrometry imaging (MALDI-MSI) allows for the acquisition of spatial distribution maps of molecular species in situ, allowing the examination of the molecular architecture in complex biological matrices, and has thus been proposed as a molecular histology tool to compliment in the study of pathophysiology [4, 5]. MALDI-MSI has demonstrated to be an effective tool for probing metabolites, lipids, and proteins in a variety of samples and tissues [4, 6–8]. When coupled with a time-of-flight mass spectrometer, MALDI-MSI covers a large mass range and has the ability to execute both intact and enzymatically digested protein analysis [9]. MALDI-MSI enables us to simultaneously detect the distribution and localization of ephrinA1, and thus elucidate its molecular partners, mediators, and targets of action [10–14]. Therefore, the primary goals of this study were: (1) to optimize an effective protocol to spatially resolve the molecular distribution of endogenous ephrinA1 using MALDI-MSI, (2) to determine and compare the anatomical expression profile of ephrinA1 in healthy control hearts versus post-acute MI, and (3) to identify some of ephrinA1 molecular partners and downstream signaling mediators.

## Materials and Methods

### Reagents

Water, acetonitrile (ACN), and trifluoroacetic acid (TFA) were Optima Grade purchased from Thermo Fisher Scientific (Hampton, NH, USA). Calibration mixture including angiotensin II, bovine insulin, polyethylene glycol 600, and MALDI matrices 2′,5′-dihydroxyacetophenone (DHA), 3,5-dimethoxy 4-hydroxycinnamic acid (sinapinic acid, SA), and α-cyano-4-hydroxycinnamic acid (CHCA) were purchased from Sigma-Aldrich (St. Louis, MO, USA). Indium tin oxide (ITO)-coated conductive glass microscope slides were purchased from Bruker Daltonics (Billerica, MA, USA). Sequencing grade trypsin was purchased from Promega (Madison, WI, USA). Accu-Edge low profile blades (4689) were purchased from VWR (Radnor, PA, USA).

### Analytical Standard Preparation

Ephrin-A1 (His tag) was purchased from NovoPro Lab (501312) and diluted to a concentration of 0.1 μg/μL in phosphate-buffered saline (PBS). For all sample slides and method development, 1 μL of the former solution was spotted on an ITO-conductive slide for MALDI analysis 1:1 with 10 mg/mL SA dissolved in 60:40 ACN:water, containing 0.07% TFA. The intact protein was analyzed using linear MALDI/TOF MS in positive ionization mode.

### Animal Use

Experimental research protocols were approved by the East Carolina University Institutional Animal Care and Use Committee (IACUC) following the guidelines of the National Institutes of Health for the Care and Use of Laboratory Animals. Male (8–12 wk-old) B6129SF2/J mice (stock #101045) were purchased from The Jackson Laboratory. All mice were housed in ventilated cages, exposed to 12 h/12 h light/dark cycle conditions, and received food and water ad libitum. The Department of Comparative Medicine at The Brody School of Medicine, East Carolina University, maintained animal care. Mice were divided in two groups: uninjured control (CTL, n = 10) and acute myocardial infarction (MI, n = 6), induced by a 4-d permanent occlusion of the left anterior descending coronary artery (LAD).

### Surgical Procedure and Tissue Collection

Mice were anesthetized with an intraperitoneal injection of Avertin (20 mg/Kg body weight, BW) and mechanically ventilated with 95% O_2_/5% CO_2_. A thoracotomy was performed and myocardial infarction was induced by occluding the LAD using an 8-0 suture. Ischemia was confirmed by visible blanching of the tissue distal to occlusion. The rib cage, muscle, and skin were closed sequentially and the animals were allowed to recover over a heating pad. Four days post-MI, animals were anesthetized with euthasol (100 mg/Kg BW) and sacrificed for tissue harvest. Whole hearts were thoroughly perfused with ice-cold PBS (10–15 mL over 2–3 min) to remove residual blood, wrapped loosely in foil, and snap-frozen in liquid nitrogen. Samples were then stored overnight at –80 °C.

### Tissue Preparation and Cryosectioning

Freshly frozen hearts were mounted to a cryostat chuck with HPLC grade water in a dry ice container to freeze and fuse the tissue in place for sectioning. Orientation of the tissue was traverse distal to ligation of LAD in infarcted hearts. Tissue was sectioned at ~10 μm thickness, thaw-mounted onto ITO-conductive microscope slides, and maintained at –20 °C. Slides were then sequentially rinsed in 70%, 90%, and 95% ethanol diluted in HPLC grade water for ~30 s each, and then placed into a vacuum desiccator containing Drierite for 20–30 min [13, 14].

### Selection of Optimal Matrix

Three matrices were tested for optimal detection of ephrinA1 standard: DHA, CHCA, and SA. All matrices were tested at 10 mg/mL in 70:30 acetonitrile:water with 0.3% TFA, 60:40 acetonitrile:water with 0.1% TFA, and 70:30 acetonitrile:water with 0.1% TFA. EphrinA1 standard was spotted with matrix 1:1 on a target plate and the intact protein signal was used to optimize the MALDI instrument parameters in positive ionization mode. The 10 mg/mL SA matrix in 70:30 acetonitrile:water with 0.3% TFA was found to provide the highest degree of detection and was selected for the rest of the study.

### Intact EphrinA1 MALDI Matrix Applications

For method development and optimization, three methods for matrix deposition were tested utilizing standards and control tissue for intact protein analysis: (1) direct delivery, (2) thin layer chromatography (TLC) sprayer (Sigma-Aldrich, Z529710, 10 mL), and (3) TM sprayer (HTX Imaging). (1) For the first experiment, 0.5 μL of the SA matrix was applied using a micropipette in multiple regions across the control tissue for intact ephrinA1 signal optimization and mixed 1:1 with the analytical standard next to the tissue for mass calibration and verification. (2) A TLC sprayer was manually used to apply SA matrix and the standard was applied near the tissue after matrix deposition. N_2_ was used at a low flow rate (< 5 psi) and psi was optimized based on the spray distribution. Two spray passes were performed before allowing the slides to completely dry. This process was repeated to add and build layers of matrix using a total of 20 mL of matrix. (3) A TM sprayer was used to apply SA matrix under the following conditions: 0.1 mL/min solvent flow rate, 10 psi N_2_ gas pressure, 30 °C nozzle temperature, 750 mm/min velocity, spacing of 2 mm, and four passes.

### Trypsin Sample Digestion

For the preparation of the trypsin solution, 20 μL of sequencing-grade trypsin (1 μg/μL) was added to 333 μL sodium bicarbonate 100 mM containing 0.01% ammonium hydroxide, 40 μL acetonitrile, and 67 μL acetic acid, for a total volume of 460 μL. The solution was prepared and incubated at 37 °C for activation prior to application on the tissue using the TM sprayer. After trypsin application, samples were incubated at 37 °C for 1, 3, and 4 h. SA matrix was applied using the TM sprayer to stop the trypsin reaction, and samples were prepared for imaging.

### Mass Spectrometry Imaging

Imaging experiments were performed using a Bruker Autoflex Speed MALDI/TOF-TOF mass spectrometer. Before matrix deposition, tissues were scanned using an Epson flatbed scanner and collected at 300 dpi for acquisition of a digital baseline image. The mass spectrometer method was set to collect 5000 spectra/spot and images. Intact protein images were collected using linear mode, and trypsin peptide images were collected using reflectron mode. Calibration was conducted using a mixture of angiotensin II, bovine insulin, PEG 600, and ephrinA1-His standard. All control and acute MI samples were imaged at 100 μm spatial resolution. Data was collected in positive ionization mode and a Nd:YAG laser repetition rate of 2000 Hz was used (50 μm laser size) during image collection.

### Data Analysis

Imaging data was processed using FlexImaging (Bruker) and the mass spectra were analyzed in FlexAnalysis (Bruker). Images were normalized using a unit vector algorithm. The murine ephrinA1 protein sequence (NCBI accession number NP_034237) was used with the MS-Digest program at http://prospector.ucsf.edu/ to predict theoretical peptide fragments from a trypsin digest of ephrinA1. The mass spectra collected were searched using Mascot Server (Matrix Science Ltd.). The search parameters included the database SwissProt with three missed cleavages, Mus musculus taxonomy, no variable modifications, average mass, and a peptide tolerance of 1.2 Da.

### Safety Considerations

Proper personal safety equipment was utilized when handling solvents and solutions. The TM sprayer is designed to contain the aerosol spray and extra precaution was considered by placing the TM sprayer in a chemical hood for ventilation. The TLC sprayer was used in a chemical fume hood to protect from aerosol inhalation.

## Results and Discussion

### Optimization of EphrinA1 MALDI Imaging Mass Spectrometry

Reliable detection of intact ephrinAl in heart tissue using MALDI/TOF MS required thorough optimization of multiple parameters. First, comparison of several MALDI matrices DHA, CHCA, and SA revealed SA to be the optimal matrix for detection of the intact ephrinA1 recombinant protein. Using positive ionization linear detection, doubly charged, protonated, and dimer forms were identified in the mass spectra (Supplementary Figure S1). To validate the selectivity of the standard, the sequence reported by the manufacturer was blasted against all databases using the National Center for Biotechnology Information (NCBI, US National Library of Medicine). Ephrin-A1 (His tag) was found to have a 349 maximum score, 94% query coverage, and a 100% identification for ephrinA1 isoform 1 precursor [Mus musculus]. Therefore, we concluded the analytical standard provided sufficient coverage and sequence overlap to accurately represent endogenous ephrinA1.

Washing has been found to significantly increase protein detection limits by reducing the suppression of ionization normally caused by lipids and small molecules [10, 11]. We modified previously reported methods [10, 11] to include sequential ethanol washes, after the tissue sections were fixed to the ITO slides, and this significantly improved the detection of ephrinA1. In fact, the intact protein was not detected in tissues when the washing step was not performed (data not shown).

**Figure 1:**
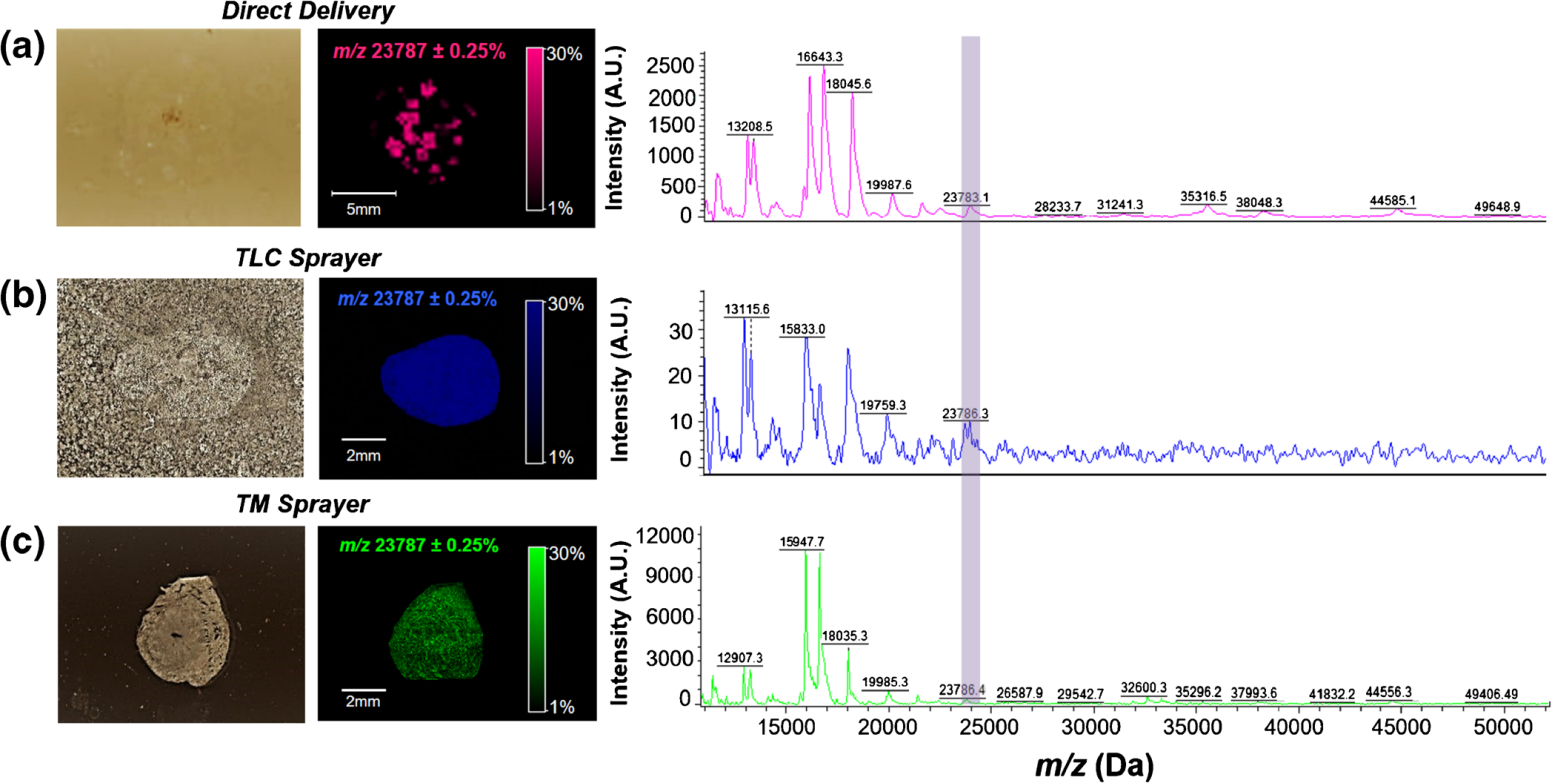
Representative pictures of chemical reconstruction images for a healthy heart using three different matrix deposition techniques: **(a)** direct delivery at *m/z =* 23783.1 (S/N 189), **(b)** TLC sprayer at *m/z* 23786.3 (S/N 10), or **(c)** TM sprayer at *m/z* = 23786.4 (S/N 97). The purple bar highlights the peak at 23.7 kDa, which represents the intact molecular ion for ephrinA1

For MALDI imaging, three different methods of matrix deposition were compared: direct delivery, TLC sprayer, and TM sprayer. The reconstructed chemical ion image for intact ephrinA1, as well as the respective mass spectra for each method, is shown in Figure 1. Intact protein signals were best detected with direct delivery and utilization of the TM sprayer. The TLC sprayer provided the lowest signal across the tissue for ephrinA1 and was the most time-consuming during the sample preparation process. Although ephrinA1 was detected with all three matrix applications, the samples prepared using the automatic TM sprayer for matrix deposition provided the most uniform reproducible signal distributions across the tissue (Figure 1c) attributable to the mechanism of the nozzle and the nature of the deposition of matrix. The TM sprayer was thus utilized to analyze cardiac tissue post-MI. We observed a decrease in the signal for ephrinA1 in non-treated tissues. However, due to the difficulties encountered with identification and mass resolution, we returned to a trypsin digestion for ephrinA1 confirmation (data not shown).

### Method Validation for Imaging of Cardiac Tissue

**Figure 2:**
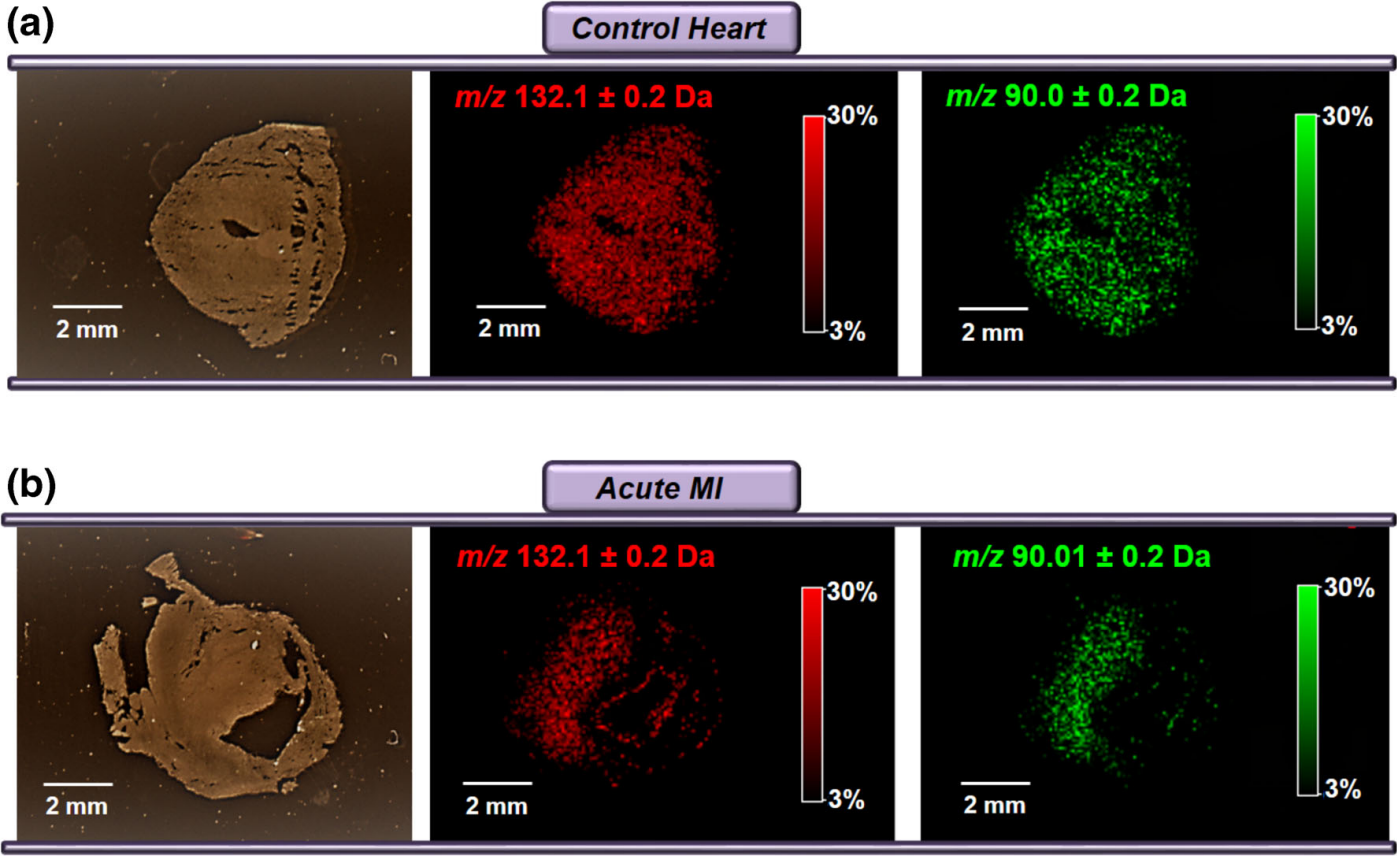
Spatial distribution of creatine (*m/z* = 132 Da, shown in red) in healthy control **(a)**, and post-MI **(b)** murine hearts. When creatine is subjected to MS/MS analysis, a primary fragment is observed at *m/z* = 90 Da, corresponding to the protonated ([M + H] ^+^) n-methylglycine (shown in green)

The molecular distribution of creatine was investigated for method validation in cardiac tissue. Creatine, which serves as a rapid energy source in cardiac tissue via the creatine/phospho-creatine system, is abundant in healthy cardiac tissue, but found to be drastically reduced in infarcted ischemic tissue [12]. The molecular ion of creatine (*m/z* = 132 Da) yields a primary fragment of *m/z* = 90 Da. Creatine and its primary fragment are shown in Figure 2, as detected in a section from a healthy control (a) and post-MI heart (b). The primary fragment of creatine presented lower signal intensity in the ischemic region post-MI. These findings are in agreement with previous reports correlating lower creatine levels with regions of necrosis, and particularly upon persisting ischemia [12]. Overall, the data support MALDI-MSI as a valid method to image murine cardiac tissue and elucidate molecular signatures post-MI.

### EphrinA1 Expression Profile in Murine Heart

**Figure 3:**
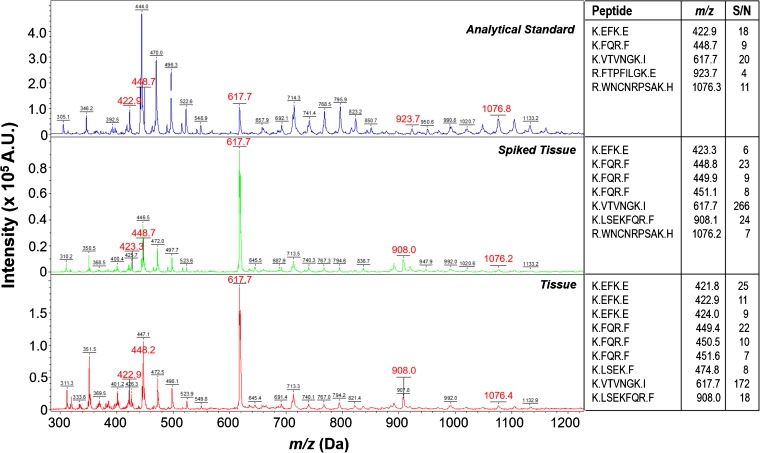
Mass spectra from ephrinA1 analytical standard (top panel), control tissue spiked with standard (middle panel), and healthy control tissue (bottom panel)

To determine ephrinA1 spatial distribution, heart sections were subjected to trypsin digestion. A 4 h incubation provided the highest number of identified tryptic fragments and sequence coverage. EphrinA1-His tag standard was also spiked in healthy control hearts prior to trypsin digestion as an analytical control, and the observed peptides were confirmed. A representative average mass spectrum is shown in Figure 3. Mascot scores for ephrinA1 were 41, 25, and 25 for the standard, spiked tissue, and tissue, respectively. The Mascot score was low for the standard due to the nature of the analysis, the main limiting factor being sample preparation for protein analysis in tissue on a surface (proteins are not fully denatured and only partially trypsinized). Taking this into consideration, the standard was treated consistently with tissue samples.

**Figure 4:**
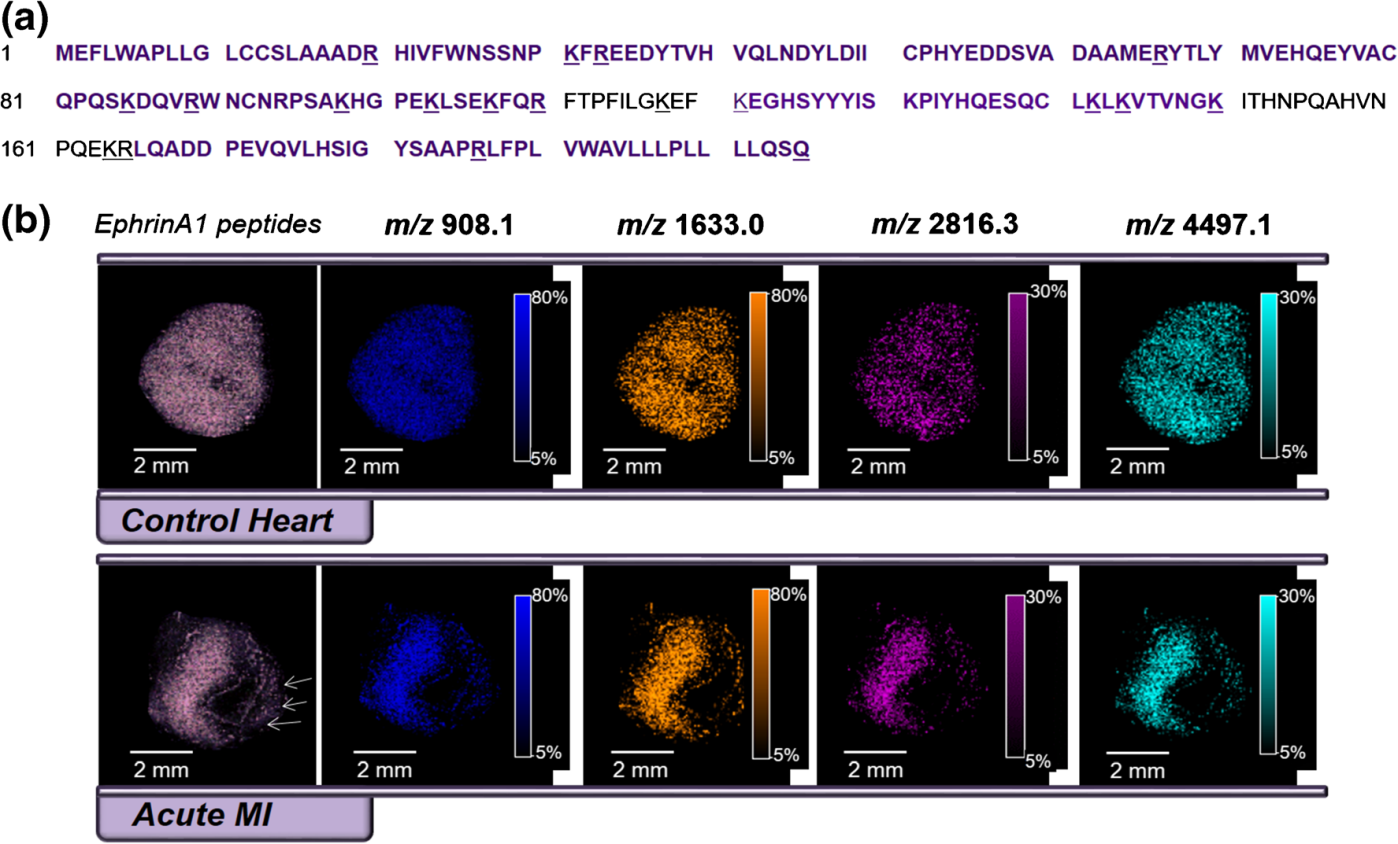
EphrinA1 in heart. **(a)** Primary sequence of ephrinA1 with trypsin cleavage sites denoted by underlined lysine (K) or arginine (R) amino acids. Murine sequence NCBI accession NP_034237, generated using the MS-Bridge program at http://prospector.ucsf.edu/ allowing for one missed cleavage. **(b)** Expression profile of trypsinized EphrinA1 fragments: matches between theoretical and detected fragments from a control, healthy heart (top), and after acute MI (bottom) are shown. Selected ephrinA1 trypsin fragments that are present in a control healthy heart (*m/z* = 908, 1632.96, 2816.25, and 4497.11 Da) but not in the region at risk from the infarcted heart are shown

EphrinA1 sequence is listed in Figure 4a. The detected tryptic peptides and reconstructed chemical images for selected ephrinA1 ions detected in the control and MI hearts are shown in Figure 4b (top and bottom, respectively). Healthy control hearts matched with 14 out of the 25 predicted weights yielding a 38% coverage, whereas the MI hearts only matched seven out of the 25 masses, yielding a 34% coverage. There were five additional peptide fragments detected in the healthy tissue that were not detected in either the standard or the MI. The reduction in the number of peptides in the MI samples is attributed to the lower abundance of ephrinA1 post-MI, in agreement with previous immunohistochemical and Western blot reports [1]. The experiment was repeated in n = 6 healthy control hearts and n = 6 MI hearts. EphrinA1 matching masses presented average protein prospector scores of 73 ± 17% and 42 ± 39% for the healthy and MI samples, respectively. There were 25 peptides specific for ephrinA1, detected with a S/N ≥ 3, and the frequency of detection per the sample is shown in Supplementary Figure S2.

### Target Cardiac Proteins Post-MI

**Figure 5:**
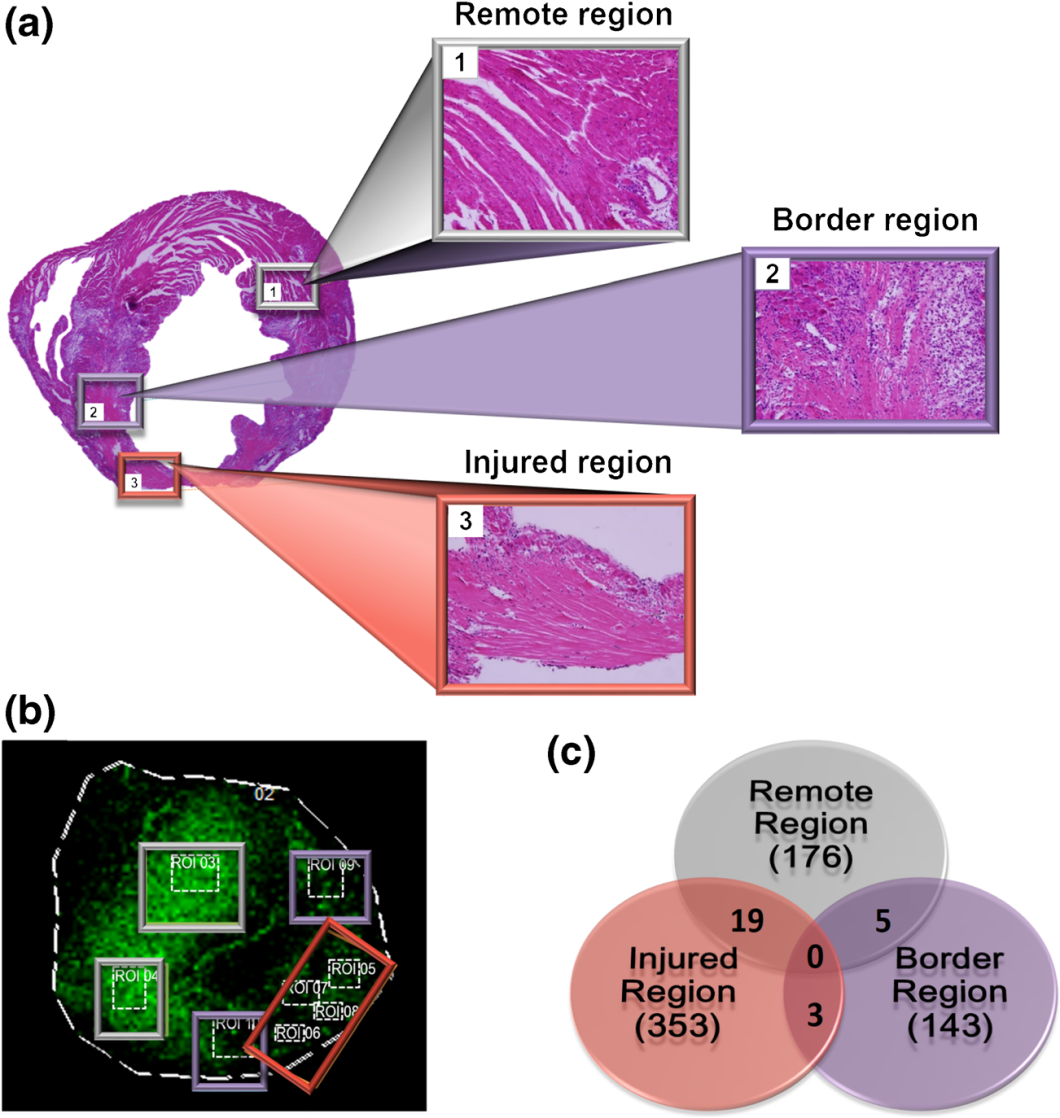
Regions of interest selected for tentative global protein identification post-MI. **(a)** Histological images (H&E) defining the remote (1), border (2), and infarcted (3) regions. **(b)** Reconstructed chemical image using *m/z* = 617.2 Da and selected regions of interest for extracting mass spectra for Mascot database searching. **(c)** Diagram showing total numbers of tentative identified proteins per region and overlapping proteins

Further analysis was conducted in post-MI tissue comparing proteins across specified regions. Three mass spectra were extracted from each defined region across the injured tissue and a Mascot search was conducted to obtain tentative protein identification. Selected regions across the infarcted heart included the remote (in the same plane as the injury but not affected by the ligation), border (the intersection of injured tissue with uninjured tissue), and injured areas shown in the H&E stain (Figure 5a). A reconstructed chemical ion image for *m/z* 617.2 shows the expression profile of ephrinA1, which was used to define the regions for MALDI-MSI extraction (Figure 5b). The Venn diagram in Figure 5c shows the overlap among the number of proteins identified within the three defined regions.

Supplementary Table 1 shows the top tentative protein identifications in healthy control versus post-MI regions of interest (remote, border, and infarct) obtained from a 4-h trypsin digest. Additional protein identification was achieved by sequence matching against Mascot, although this was not further validated with a secondary technique. The proteins depicted in Supplementary Table 1 warrant further investigation to elucidate the role of endogenous ephrinA1 in the cardiomyocyte, as well as its molecular targets upon acute MI. Specifically, pathways involved in mitochondrial energetics and autophagic flux are of great interest to understand the molecular mechanisms by which ephrinA1-Fc mediates preservation of myocardial tissue integrity and cardiac function upon MI.

Using the functional annotation tool (DAVID 6.8), we identified 18, 30, 21, and 61 clusters in the healthy remote, border, and infarct regions, respectively (Supplementary Table 1). As expected in healthy control hearts, the four clusters with the highest enrichment scores (ES) were identified with the mitochondria, nucleus, RNA processing, and the innate immune response. In the remote region of the infarcted LV, however, the highest ES scores were displayed by proteins associated with altered enzymatic activity such as lyases, isomerases, and GTPases, indicative of increased remodeling activity [15]. Interestingly, the annotation clusters identified in the border region of the infarcted LV showed high ES for ribosomal proteins, DNA binding, and mRNA processing. These data suggest high transcription and translational activity, which is expected during the healing and regeneration process post-MI (i.e., increased protein turnover) [16, 17]. The fourth annotation cluster with the highest ES in the border zone was associated with the defense response, in accordance with inflammation being a fundamental process during the early stages of post-infarction myocardial remodeling [18, 19]. These are activated after ischemia and play an essential role in several pathways to facilitate clearance of apoptotic cells and necrotic tissue, as well as to increase metabolic activity to compensate for the oxygen deprivation and reduced substrate availability. Finally, annotation clusters from the infarcted LV proteins included mitochondrial and metabolic enzymes, lipoproteins, phagocytic vesicles, and proteins involved in redox processes.

## Conclusions

The protocol described herein offers the ability to resolve the anatomical distribution of endogenous myocardial ephrinA1 and additional proteins of interest using MALDI imaging. To our knowledge, no other technique other than immunohistochemistry has provided spatially resolved images of ephrinAl expression in heart tissue. MALDI-MSI provides an alternative and significantly more powerful method that allows for customized high-resolution images of ephrinA1. This can be achieved by an optimal tissue sample preparation and optimization of experimental and technical parameters, including tissue digestion methods, matrix selection, matrix deposition, and spatial resolution. The optimization of this protocol would potentially aid in the development of a new platform for the study of molecular signatures of ischemic injury in the myocardium.

## Electronic supplementary material

ESM 1. (DOCX 195 kb)
