# Supplementary material for: Anatomical-Molecular Distribution of EphrinA1 in Infarcted Mouse Heart Using MALDI Mass Spectrometry Imaging
Source: J Am Soc Mass Spectrom. 2018 Jan 5;29(3):527–34. doi: 10.1007/s13361-017-1869-7 (PMC5838209; doi:10.1007/s13361-017-1869-7)
Supplement: Supplementary file 1 — (DOCX 195 kb) [file 13361_2017_1869_MOESM1_ESM.docx]

**Supplemental: Anatomical - Molecular Distribution of EphrinA1 in Infarcted Mouse Heart using MALDI Imaging Mass Spectrometry**

Stephan Lefcoski^1*^, Kimberly Kew^2*^, Shaun Reece^1*^, Maria J. Torres^3^, Justin Parks^1^, Sky Reece^1^, Lisandra E. de Castro Brás^1^, and Jitka A. I. Virag^1†^

^1^Department of Physiology, Brody School of Medicine, ^2^Department of Chemistry, East Carolina University, ^3^East Carolina Diabetes and Obesity Institute, Greenville, NC


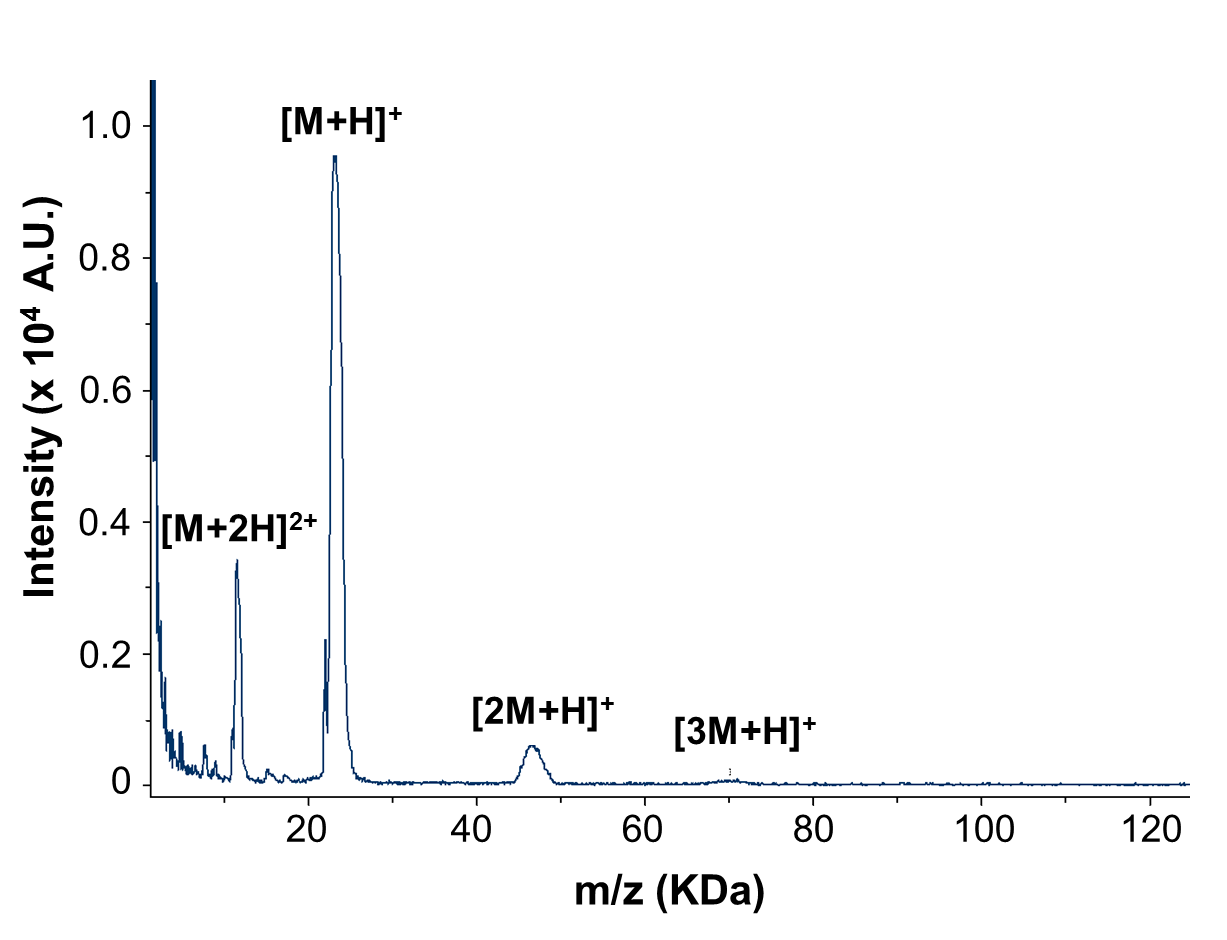


**Figure 1S: Mass spectra for EphrinA1-His standard as intact protein analysis.**

**
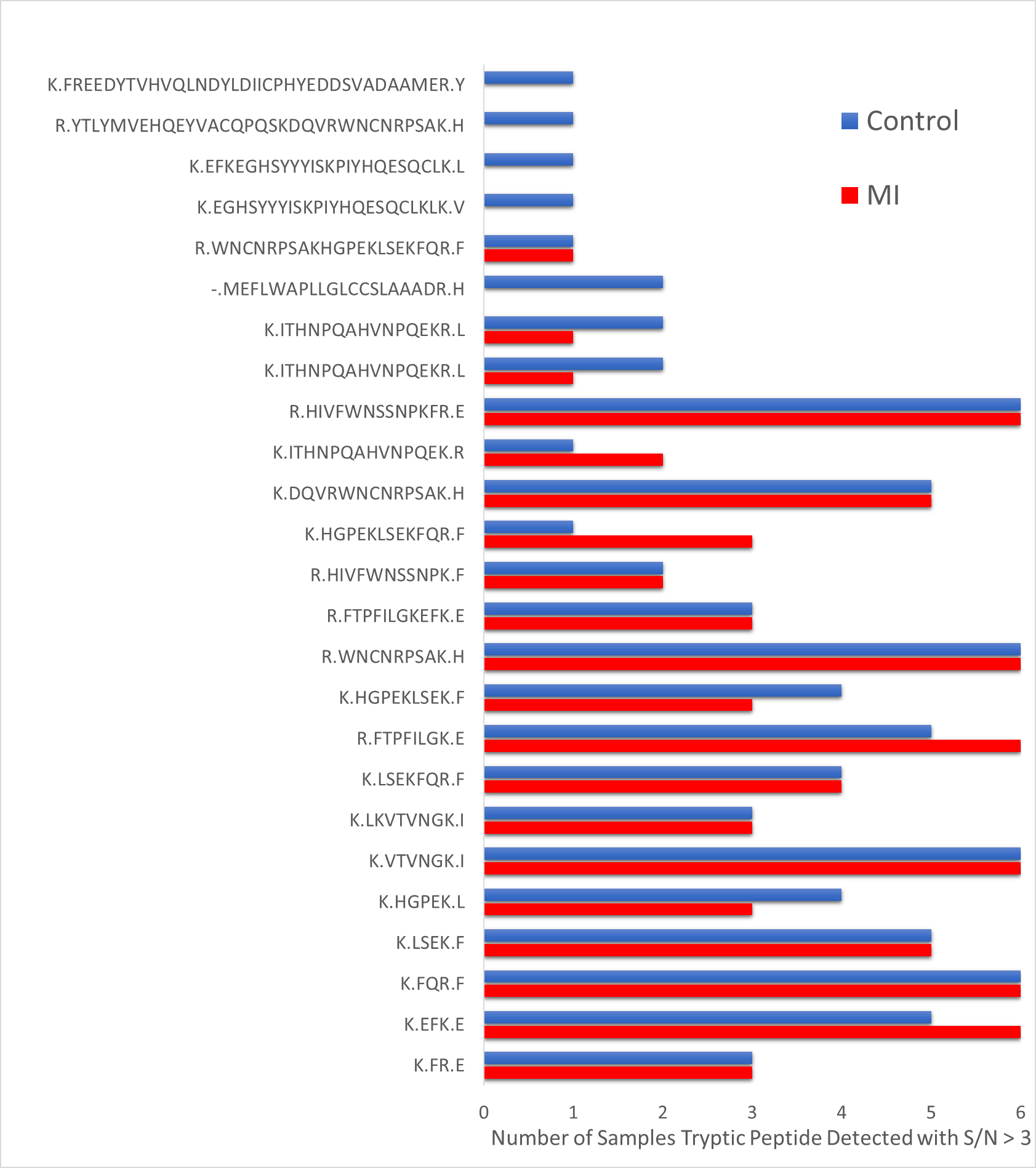
**

**Figure 2S: Frequency distribution for tryptic peptide detection in healthy and MI heart tissues with n=6 per group.**

**Table 1S: Tentative protein identifications in healthy heart (control) and post-MI regions of interest (remote, border and infarct). Sections underwent trypsin digest for 4 hours.**

| ***Regions of interest*** | **Enrichment Score** | **Pathway** | **Proteins** |
| --- | --- | --- | --- |
|  |  |  |  |
| ***Control*** | **1.46** | **mitochondria** | **ATP5E, COX7A2, ATP5F1, COX7A2L, CDK5, COX7A2L, HIGD2A, GPX4,** |
| **Total clusters 18** |  |  | **CISD2, MRPS28, MCL1, GDAP1, COX7A2, MCL1, GDAP1, ALKBH3, FAM32A** |
|  |  |  |  |
|  | **1.28** | **nucleosome and methylation** | **UTP3, HIST1H2AF, HIST2H2AC, HIST1H2AH, H1FOO, CRH, PFN1, MCL1,** |
|  |  |  | **NSD1, SRSF1, GPSM1, PNN** |
|  |  |  |  |
|  | **1.17** | **RNA processing** | **SRSF1, YTHDC1, RBMY, RBM28, PNN, PPIG, LARP7, RSL1D1, JAKMIP1** |
|  |  |  |  |
|  | **1.01** | **innate immune response** | **DEFB29, DEFB18, DEFB22, RDN, COL4A1, PTN, WNT11, MANF** |
|  |  |  |  |
| ***Remote*** | **2.34** | **lyases activity** | **PCBD2, MLYCD, NEIL3, AMD1, AMD2, AUH** |
| **Total clusters 30** |  |  |  |
|  | **2.14** | **isomerases** | **PPIE, PPID, TTC9, PIN4, PBLD2, TRUB1** |
|  |  |  |  |
|  | **1.74** | **ribosome and mitochondria** | **QRDL, MRPL52, MRPL21, MLYCD, MTG1, MRPS12, MRPL19, MRM2, SCO2, AUH, GLRX2** |
|  |  |  | **RPS27, RPS27L, SQRDL, PCBD2, RAF1, COMT, CBR2, KRAS, COA5, RAB35, PPID, COA7, RHOA,** |
|  |  |  | **MRPL52, RPS27, MRPL21, MRPS12, MRPL19, RPS27L** |
|  |  |  |  |
|  | **1.41** | **GTP-binding** | **RSG1, GIMAP4, RABL3, KRAS, MTG1, RAB35, RASL10B, RHOA, GCH1, OLA1,** |
|  |  |  | **CNP, SMC2, CHST4, CNP, CDK9, UBE2QL1, CDKL3, PRKAR2B, REP15, WNT6, TRAPPC3L, PPIE** |
|  |  |  |  |
| ***Infarct*** | **8.56** | **mitochondria** | **HCCS, AURKAIP1, MIGA1, GOT2, NDUFS6, CISD2, DNAJC15, DYNLL1, MRPL36, PDHA1, HADH,** |
| **Total clusters 61** |  |  | **MRPL53, NMNAT3, FECH, CYP11A1, ACADS, PLD6, HRK, PFDN2, SLC25A33, UQCRH, G0S2,** |
|  |  |  | **MRPL45, FASTKD5, GATB, TUFM, TXN2, MTX1, ECHS1, PTPMT1, MRPL20, MRPL15, MRPL18,** |
|  |  |  | **SLC25A42, AGK, ETFB, ECI1, NDUFA2, SIRT4, MPC1, RAF1, AK4, TRNT1, PRELID1, RAB32, UCP2,** |
|  |  |  | **L2HGDH, COA7, ENDOG** |
|  |  |  |  |
|  | **4.83** | **dehydrogenases and monooxygenases** | **TUFM, NIT2, VAPB, MME, ECHS1, HSPA1B, PRDX1, PKM, GOT2, GPX1, CISD2, TMED4, DYNLL1,** |
|  |  |  | **RPL8, RHOA, RPL3, TMED10, PDHA1, HADH, GAPDH, ETFB, GSTA1, ECI1, RAB8A, NDUFA2,** |
|  |  |  | **ACADS, AK4, SLC9A3R1, LIN7A, UQCRH, TXNDC5, HAO2, RAB22A, CSTB** |
|  |  |  | **UGDH, RRAD, BLOC1S6, ENO2, GSTA2, KHK, KRT18, HAO2, RPS12** |
|  |  |  |  |
|  | **3.31** | **GTP signaling, phagocytic vesicles, and lipoproteins** | **RAB8A, RAB8B, RAB33A, RAB32, DIRAS2, RAB18, PTP4A3, RAB37, RAB22A, RHOA, GNG2,** |
|  |  |  | **RAB10, GNG4, YKT6, FBXL2** |
|  |  |  | **HCCS, GABARAPL2, HPCAL4, MME, CDC42SE1, ARF6, KCNIP3, GOLGA7, FAM49B, CHIC2, LIME1,** |
|  |  |  | **WNT8A, CIB1, GFRA3, TUFM, GIMAP4, RRAD, AK4, SRPRB, GCH1, RABIF** |
|  |  |  | **HSPA1B, MRPL20, RPL8, MRPL36, ENO2, RPL3, MRPL18, GEMIN2, TGFBR1, VTI1B, H2-AB1,** |
|  |  |  | **ANXA5, TRNT1, RASSF5, MAP3K15, CSTB, VAMP3, FABP2, ELP4, PSTK, GUK1** |
|  |  |  |  |
|  | **3.05** | **redox processes** | **STEAP3, STEAP4, LDHB, NDUFA2, LDHA, SDR39U1, CYP11A1, ACADS, TXN2, UGDH, PRDX1, ADI1,** |
|  |  |  | **NDUFS6, GPX1, L2HGDH, UQCRH, FMO2, HAO2, TXNRD1, GPX8, PDHA1, ALKBH3, HADH, DR9C7,** |
|  |  |  | **ETFB, GAPDH** |
|  |  |  |  |
| ***Border*** | **10.04** | **Ribosomal proteins** | **RPL18, MRPL52, RPL36A, MRPS33, RPL26, RPS27L, RPL37, RPL24, RPL38, RPL39, MRPL30, RPS8,** |
| **Total clusters 21** |  |  | **RPL29, RPS26, RPL41, RPL32, RPL32-PS, RPL21, RPS14, FAU, RPS13, RPL7A, MRPL33, RSL24D1** |
|  |  |  | **SNRPD1, ZCCHC17, SNRPE, EIF3D, HMGB1, MANF, H2AFZ, H3F3A, KRAS, AIF1L, PRPF4B, UBE2L3,** |
|  |  |  | **HIST1H1T, SPCS1, NKIRAS1** |
|  |  |  |  |
|  | **6.12** | **DNA binding and nucleosome** | **RPL18, NKAP, PRPF4B, AURKAIP1, RBM7, RPS19BP1, CBX5, EBNA1BP2, HIST1H2BM, H2AFV,** |
|  |  |  | **MAK16, ELOF1, RBM8A, DNAJC8, H2AFZ, HIST3H2BA, MRPL33, CABLES1, SPATA24, ARGLU1,** |
|  |  |  | **MRPL52, POLR1D, ZCCHC17, HIST1H1T, RPS14, LLPH, UBD, RPS13, ING5, THAP7, ING2, POLR2K,** |
|  |  |  | **FCF1, RPS26, HIST1H4A, RSL24D1, TCEA2, RPL7A, FAM32A, ARL6IP4, HIST1H2BB, HIST1H2BH,** |
|  |  |  | **MRPL30, UQCC2, SRSF5, RPL21, RSRC1, NOP16, HIST1H3A, HIST1H3B, H3F3A, MPHOSPH6,** |
|  |  |  | **SNRPD1, SOX16, SOX19, HIST1H1B, MXD4, SNRPE, PRPF38A, HMGB1, HMGB3, TCEA3, TNP1,** |
|  |  |  | **UBE2L3, SNURF, SYF2, PRM1, H3F3C, PRM2, SNRNP27, S100A5, RPS27L, MANF, SERF2, RPS8,** |
|  |  |  |  |
|  | **3.66** | **mRNA processing** | **HIST1H2BB, HIST1H1B, HIST1H2BH, TNP1, HIST1H2BM, HIST1H1T, H2AFV, HIST1H4A, HIST1H3A,** |
|  |  |  | **H2AFZ, HIST1H3B, H3F3A, PRM1, H3F3C, PRM2, HIST3H2BA, HMGB1, THAP7, HMGB3, CBX5,** |
|  |  |  | **LLPH, POLR1D, PRPF4B, SNRPD1, ING5, KRAS, MXD4, TCEA3, SOX16, TCEA2, SOX19, SPATA24,** |
|  |  |  | **ING2, RPL26, UBE2L3, TMA7,CXCL14, RBM8A, TCEA2, MPHOSPH6, UBD, RPL29, ING2, POLR2K,** |
|  |  |  |  |
|  | **3.06** |  | **SRSF5, PRPF4B, RBM8A, RSRC1, SNRPD1, SYF2, ARL6IP4, SNRNP27, SNRPE, PRPF38A, POLR2K,** |
|  |  |  | **DNAJC8, RBMX2, AURKAIP1, CCDC12, RBMX2, THAP7** |
